# Optimal migration energetics of humpback whales and the implications of disturbance

**DOI:** 10.1093/conphys/cov001

**Published:** 2015-03-09

**Authors:** Janelle E. Braithwaite, Jessica J. Meeuwig, Matthew R. Hipsey

**Affiliations:** 1 School of Animal Biology (UWA Oceans Institute), Faculty of Science, University of Western Australia, Crawley, WA 6009, Australia; 2 The Centre for Marine Futures (UWA Oceans Institute), Faculty of Science, University of Western Australia, Crawley, WA 6009, Australia; 3 School of Earth and Environment (UWA Oceans Institute), Faculty of Science, University of Western Australia, Crawley, WA 6009, Australia

**Keywords:** Anthropogenic disturbance, bioenergetics, migration

## Abstract

Whales migrate long distances and reproduce on a finite store of energy. Budgeting the use of this limited energy reserve is an important factor to ensure survival over the period of migration and to maximize reproductive investment. For some whales, migration routes are closely associated with coastal areas, exposing animals to high levels of human activity. It is currently unclear how various forms of human activity may disturb whales during migration, how this might impact their energy balance and how this could translate into long-term demographic changes. Here, we develop a theoretical bioenergetic model for migrating humpback whales to investigate the optimal migration strategy that minimizes energy use. The average migration velocity was an important driver of the total energy used by a whale, and an optimal velocity of 1.1 m s^−1^ was determined. This optimal velocity is comparable to documented observed migration speeds, suggesting that whales migrate at a speed that conserves energy. Furthermore, the amount of resting time during migration was influenced by both transport costs and feeding rates. We simulated hypothetical disturbances to the optimal migration strategy in two ways, by altering average velocity to represent changes in behavioural activity and by increasing total travelled distance to represent displacement along the migration route. In both cases, disturbance increased overall energy use, with implications for the growth potential of calves.

## Abstract (precis)

Whales migrate long distances and reproduce on a finite store of energy. We model the energy use of migrating humpback whales and find optimal strategies exist for animals to conserve energy. Human disturbance may increase the overall energy use of whales if they cause deviations from these optimal migration strategies.

## Introduction

All animals require energy to sustain life. Energy consumed from the environment is used to maintain basic functioning, to support daily activities and to reproduce. Maximizing the efficiency of energy intake and subsequent use drives foraging patterns (Perry and Pianka, 1997), life-history traits, such as breeding strategy (Gadgil and Bossert, 1970) and hibernation (Nedergaard and Cannon, 1990; French, 1992), and natural selection (Lotka, 1922). Fundamentally, the survival and reproductive success of a population requires sufficient energy intake and efficient energy use. Any natural or human-induced disruptions to these processes, such as a reduction in food availability or an increase in energy demands, may have long-term implications for population survival and growth (Croxall *et al.*, 1999; Oro and Furness, 2002; Rode *et al.*, 2010).

The energetic repercussions of human disturbance therefore provide an important consideration for conservation management (Cooke *et al.*, 2012, 2013), though it has largely been unexplored as a management tool for humpback whales (*Megaptera novaeangliae*), whose migration routes can be ­disturbed by coastal developments. For example, in northwest Australia the current industrial developments supporting the expansion of offshore oil and gas and onshore mining activities (Department of Mines and Petroleum, 2014) coincide with known humpback whale migration routes and resting areas (Chittleborough, 1953; Jenner *et al.*, 2001; Braithwaite *et al.*, 2012) as they journey between breeding areas located off the northwest coast of Australia and feeding grounds in the Southern Ocean (Chittleborough, 1965). While no behavioural changes associated with human disturbance have yet been observed in this population, similar threats have led to changes in the normal behaviour of whales in other populations. Increased shipping, physical offshore infrastructure, dredging and the noise pollution associated with these industries have caused, for example, increases in swimming speed (Baker and Herman, 1989; Au and Green, 2000), avoidance behaviour (Maybaum, 1993; McCauley *et al.*, 2000; Gordon *et al.*, 2003), entanglement (Cassoff *et al.*, 2011; Meyer *et al.*, 2011) and longer singing times for courting males (Miller *et al.*, 2000; Fristrup *et al.*, 2003). Of those disturbances that are not fatal to migrating humpback whales, many are likely to generate energy costs. It is currently unclear how disturbances to humpback whales along the migration route impact the energy use of these whales or what the long-term consequences of potential energy disruptions might be to achieving migration and breeding. For the purposes of this study, we define disturbance as an activity that changes the normal behaviour of animals.

Organisms allocate energy to processes such as basal metabolism, thermoregulation, activity, reproduction, storage and growth. The division of energy between these depends on the sex and life stage of the animal and on environmental conditions (Kooijman, 2009). For example, a pregnant female will require more energy for reproduction, while an animal in cold environments may need to thermoregulate by increasing its metabolic rate. The optimal allocation of resources is an important component to fitness and selective pressure, because reproductive success requires enough energy investment at the right time (Perrin and Sibly, 1993). When energy intake is limited, increased demand for one process may compromise the energy available for others, potentially reducing reproductive effort. This can be both natural and anthropogenic. For Magellanic penguins (*Spheniscus magellanicus*), the locations of highly productive feedings areas change with oceanographic conditions; increased foraging trip distances and durations cause high locomotive costs, negatively affecting breeding success (Boersma and Rebstock, 2009). In oystercatchers (*Haematopus ostralegus*), the introduction of experimental disturbance to parental foraging reduced the amount of food delivered to the chicks, even when the amount of food collected remained similar, suggesting that the extra energy demand for activity reduced the allocation to reproduction (Verhulst *et al.*, 2001). In more general terms, it is apparent that changes in the environment, including the introduction of human disturbance, can alter energy allocation, potentially to the detriment of reproduction and population resilience.

There are two general strategies available to an animal to supply the energy needed for reproduction: income and capital breeding (Jonsson, 1997; Stephens *et al.*, 2009). For income breeders, energy replenishment occurs concurrently with breeding, while capital breeders are fasting, supplying the energy for reproduction from stores accumulated previously (Jonsson, 1997; Stephens *et al.*, 2009). For capital breeders, reproductive effort and success is related to maternal mass, with larger females positively influencing traits such as pregnancy occurrence (Boyd, 2000), lactation length (Wheatley *et al.*, 2006), milk delivered (Crocker *et al.*, 2001), offspring weaning mass (Derocher and Stirling, 1990; Bowen *et al.*, 2001a, 2006; Wheatley *et al.*, 2006) and fecundity (Lockyer, 1986; Klanjscek *et al.*, 2007; Miller *et al.*, 2011), whereas the reproductive traits of income breeders are more dependent on the fluctuating environment and food availability (Bowen *et al.*, 2001b). Reproductive success is therefore related to the energy available to be invested into offspring. However, the stored energy of capital breeders is finite, meaning that any increases in energy demands for other processes will reallocate energy away from breeding, with repercussions on reproductive success. Given that these energy stores cannot be replenished until after the breeding season, capital breeders are also vulnerable to exhaustion of their energy reserves before foraging grounds are reached (Jonsson, 1997). If human disturbance affects energy use during the breeding ­season, it would have implications for the reproductive success, growth rates and, ultimately, survival.

Humpback whales, like many large whale species, are at the extreme end of capital breeding. They annually migrate thousands of kilometres between foraging grounds in the polar regions and breeding grounds in the tropics. During this migration the adults are not feeding and therefore rely on energy stores to fuel the 8–9 month journey. In addition, other energetic costs associated with reproduction, breeding and lactation, are incurred over this same time period (Chittleborough, 1958). Increases in energy expenditure therefore have the potential to impact the reproductive success of these animals and their ability to complete the migration cycle before stores are depleted. Disturbance from human activities, such as the presence of boat traffic and seismic survey noise, has been documented to change the behaviour and activity of large whales (e.g. avoidance, Corkeron, 1995; Stamation *et al.*, 2009; increased song duration, Miller *et al.*, 2000; and increased swimming speed, Baker and Herman, 1989; Au and Green, 2000), particularly groups containing females and calves (Lundquist *et al.*, 2008; Stamation *et al.*, 2009). Higher activity levels may impact reproduction by diverting the limited energy available away from lactation in females or growth in calves. The close association of Australian humpback whales with the coastline during southbound migration exposes whales to high levels of human activities, increasing the likelihood of disturbance on breeding grounds and during their journey towards the Southern Ocean feeding grounds. In Australia, humpback whales are also known to aggregate in sheltered coastal areas to rest during their journey (Chittleborough, 1953; Jenner *et al.*, 2001). The high densities of whales in these areas, where groups contain a high proportion of females with calves (McCauley *et al.*, 2000; Braithwaite *et al.*, 2012), also increases the likelihood of encounters between whales and human activities and, thus, the number of whales that may be affected. While local disturbances to behaviour may seem minor, the costs of repeated disruptions may accumulate over the long journey, collectively having a major impact on the energy stores of the whales. Furthermore, whales may be more vulnerable to increases in energy use during those periods of resting, where energy conservation appears to be important in habitat selection (Whitehead and Moore, 1982; Felix and Botero-Acosta, 2011).

In this study, we develop a bioenergetic model for migrating humpback whales. This model is first used to understand migration strategies under varying swimming speeds and resting vs. travelling scenarios, where the optimal strategy is defined as that which minimizes energy expenditure. We focus on lactating females in the optimal migration simulations, because they expend more energy compared with other life stages or sexes (Lockyer, 1986; Fortune *et al.*, 2013). We then simulate disturbance to whales through two drivers: increased swimming speed to mimic increased activity, and increased distance travelled to mimic diversion from the typical migration route through displacement. Changes to the growth rates of calves are assessed under these two disturbance scenarios. Energy is a valuable and finite resource for humpback whales, required to support survival and reproduction. It is therefore crucial to understand how disturbances along the migration route may disrupt optimal energy use and impact fitness and reproduction.

## Materials and methods

We modelled the period of migration between departure from the calving grounds until arrival at the foraging ground, ­covering a period of ∼3 months (Chittleborough, 1965; Jenner *et al.*, 2001). For humpback whales migrating along the west Australian coast, this equates to a journey of ∼8500 km over 90 days, during which a young calf accompanies a female. This 90 day time period reflects a very early period in the life of calves, which are likely to be only a few weeks old when migration begins, given the temporal movements recorded for this population (Jenner *et al.*, 2001).

The energy use of a migrating humpback whale was allocated among maintenance, activity, lactation and growth, depending on the age and sex of the whale. An adult male uses energy for maintenance and activity, an adult female uses additional energy in lactation, and a calf uses energy for growth (Winship *et al.*, 2002; Fortune *et al.*, 2013). The calculations for each of these components are outlined in the f­ollowing sections.

Thermoregulation is not included in this model because it is likely not to be a significant energy drain for migrating humpback whales; a change in metabolic rate for ­thermoregulatory purposes is only relevant outside an animal's thermoneutral zone, and within this zone physiological adaptations are enough to maintain a constant body temperature (Willmer *et al.*, 2005). Evidence from theoretical modelling suggests that the lower critical temperature for large cetaceans, including their calves, is much lower than the minimal seawater temperature (Lavigne *et al.*, 1990). The upper critical temperature of large whales has not been identified. However, the water temperatures experienced remain lower than core body temperatures, so heat will continually be lost from the animal. Furthermore, physiological and behavioural mechanisms exist to regulate the efficiency of heat lost (Pabst *et al.*, 1999). We therefore assume that humpback whales do not reach their upper critical temperature and stay within their thermoneutral zone during migration.

Model parameters presented in the following sections are summarized in Table 1.

**Table 1:** Model parameters with corresponding values used in simulations

Parameter	Abbreviation (units)	Value	Source
Basal metabolic rate	BMR (kJ day^−1^)	292.9$M^{0.75}$	(Kleiber, 1975)
Ratio of active to passive drag	λ	0.7	(Hind and Gurney, 1997)
Assimilation efficiency	ϵ_A_	0.2	(Fish, 1996)
Propeller efficiency	*ϵ*_P_	0.8	(Fish, 1996)
Mass density of fluid	ρ (kg m^−3^)	1027	Standard for sea water
Surface area	*S* (wetted; m^2^)	0.045$M^{0.696}$	(Ryg *et al.*, 1993)
Drag coefficient	*C*_d_	0.0034 (minke whale)	(Hind and Gurney, 1997)
		0.00306 (sperm whale)	(Miller, 2004)
		0.0026 (fin whale)	(Miller, 2004)
		0.0029 (killer whale)	(Fish, 1998)
		0.003	Used is this study, mean for whale values
Mass of calf growth	d*M* (kg)	8850	Over 10.5 months, see text
Proportion of lipid growth	*P*_lip_	0.22	See main text
Proportion of water in lean tissue	*P*_w_	0.672	(Fortune *et al.*, 2013)
Energy density of lipid	ED_lip_ (kJ kg^−1^)	39 300	(Schmidt-Nielsen, 1997)
Energy density of protein	ED_pro_ (kJ kg^−1^)	18 000	(Schmidt-Nielsen, 1997)
Proportion of lipid in milk	*M*_lip_	0.438	(Oftedal, 1997)
Proportion of protein in milk	*M*_pro_	0.091	(Oftedal, 1997)
Swimming velocity	*V* (m s^−1^)	0.5–1.0 (slow)	(Jenner and Jenner, 2011)
		1.0–2.5 (medium)	
		>2.5 (fast)	

See main text for parameter definitions.

### Basal metabolic rate

The energy required to support the basic functioning of an organism at rest is generally defined as the basal metabolic rate (BMR). Kleiber (1975) identified an allometric relationship between BMR and body mass among animals, calculated as $E_{\mathrm{BMR}}=70M^{0.75}$, where $E_{\mathrm{BMR}}$ is measured in kilo­calories per day and mass (*M*) in kilograms. This relationship describes interspecific variation, and its application to calculating intraspecific variation is unclear (Boyd, 2002), because the scaling of intraspecific metabolism is highly variable (Glazier, 2005). However, as no intraspecific relationship has been established for humpback whales, we used the standard Kleiber (1975) equation in our models (Table 1). The $E_{\mathrm{BMR}}$ of a calf over the 90 day period modelled here was estimated to be twice that of an adult to account for the elevated ­metabolic rates of immature animals (Lavigne *et al.*, 1986; Worthy, 2001).

### Activity/cost of transport

The main activity of migrating humpback whales is travelling (swimming). Thus, we generalized energy expenditure of all activity as the cost of transport (COT). Resting and travelling behaviours were categorized based on swimming speed, with resting whales swimming at slower speeds (0.5–1 m s^−1^; Jenner and Jenner, 2011) than travelling whales (ranging between 1 and 4 m s^−1^; Chittleborough, 1953).

We calculated the cost of a whale swimming through the water based on the amount of energy required (*E*_COT_, in joules per second) to overcome the drag forces (*D*, in newtons) of actively swimming at a constant speed (*V*, in metres per ­second; Hind and Gurney, 1997):

(1) $$E_{\mathrm{COT}}=D\times V$$

where drag is calculated as:

(2) $$D=\;\frac{1}{2}\;\rho SC_{d}V^{2}$$

with ρ being the density of sea water (in kiograms per cubic metre), *S* the wetted surface area (in square metres) and *C*_d_ the drag coefficient. The wetted surface area was calculated using a general scaling relationship between mass and surface area described for marine mammals (Ryg *et al.*, 1993; Table 1). The drag coefficient is assumed to be constant across velocities, because the large body of a whale produces a high Reynolds number (turbulent) flow (Batchelor, 1967; Hind and Gurney, 1997). Equation 2 is derived for a passive object moving through a medium, whereas whales are active swimmers and will therefore be creating extra drag through body movements. The equation is therefore scaled by the ratio of active to passive drag (λ), to account for this extra drag (Hind and Gurney, 1997). The energy required to swim will also depend on the efficiency with which metabolized energy is converted into mechanical work (aerobic efficiency, ϵ_A_) and the efficiency with which muscle movements are converted into forward motion (propulsive efficiency, ϵ_P_; Fish, 1996; Hind and Gurney, 1997). For cetaceans, which are lift-based swimmers, aerobic efficiency typically reaches 20%, while propulsive ­efficiency is at least 80% (Fish, 1996). The total energetic cost of transport for a migrating whale is therefore calculated as:

(3) $$E_{COT}=\left(\frac{\lambda }{2\epsilon_{A}\epsilon_{P}}\right)\rho SC_{d}V^{3}$$

Water motion also influences the cost of transport, with the aligned flow reducing *E*_COT_ and opposing flow increasing it. For the purpose of this research, we assumed that fluctuations in *E*_COT_ due to water motion were equalled out over the migration cycle, and thus considered constant, though it could be accounted for by modifying the net migration velocity for studies over shorter time scales.

### Lactation

The energy demands of a migrating female with a calf include lactation alongside BMR and COT, to produce enough milk to support the maintenance and growth of the calf. The energy required for lactation depends upon the energy content in the milk produced, which in turn is a function of the protein and lipid content. The metabolic demand of producing a quantity of milk in a given time (*E*_LAC_, in kilowatts) can be described as:

(4) $$E_{\mathrm{LAC}}=M_{m}\times [(M_{\mathrm{lip}}\times ED_{\mathrm{lip}})+(M_{\mathrm{pro}}\times ED_{\mathrm{pro}})$$

where *M*_m_ is the mass of milk transferred in a given time (in kilograms per second), *M*_lip_ the proportion of lipid in the milk and *M*_pro_ the proportion of protein in the milk; ED_lip_ and ED_pro_ are the energy densities of lipid and protein, respectively (in kilojoules per kilogram; see ‘*Growth*’ section).

Cetacean milk generally contains water, protein and fat, and these constituent concentrations change over time (Oftedal, 1997). For humpback whales, numbers are provided only for 4–7 months postpartum, so we assumed these values (42.9% water, 43.8% fat and 9.1% protein) for our model. The quantity of milk required needs to meet the energy needs of the calf and offset the assimilation efficiency of digesting (see ‘*Growth*’ section). Thus, 110% of the total energy requirements of the calf needs to be provided in the milk.

The daily energy requirement of a lactating female (*E*_FEMALE_, in kilojoules per day) is summarized as:

(5) $$\begin{array}{l} E_{\mathrm{FEMALE}}=E_{\mathrm{BMR}}+E_{\mathrm{COT}}+E_{\mathrm{LAC}}=292.9M^{0.75}+\left(\frac{43,200\lambda }{\epsilon_{A}\epsilon_{P}}\;\right)\\ \;\;\;\rho SC_{d}V^{3}+M_{m}\times [(M_{\mathrm{lip}}\times ED_{\mathrm{lip}})+(M_{\mathrm{pro}}\times ED_{\mathrm{pro}})]\;\; \end{array}$$

Initial female mass was set at 30 000 kg, derived from the length-to-weight relationship of Lockyer (1976) for a female of length 13 m, and the energy demand of lactation was computer based on the growth rate of a calf, as discussed in the next section.

### Growth

A calf requires sufficient energy to support maintenance (BMR), transport (COT) and growth. Humpback whale calves measure ∼4.3 m at birth and ∼8.8 m at weaning (Boyd *et al.*, 1999). Using the Lockyer (1976) species-specific length-to-weight relationship, this equates to an initial mass of ∼1200 kg and weaning mass of ∼10 050 kg. Thus, ∼8850 kg of mass is accumulated through growth over a 10–11 month weaning period (Boyd *et al.*, 1999), averaging a daily growth of ∼28 kg of mass. According to the allometric relationship between sculp (blubber and skin) and body mass, defined by Ryg *et al.* (1993), ∼1644 kg of mass growth will be stored as blubber, using sculp as a proxy for blubber mass. The remaining 7206 kg of mass growth is categorized here as lean tissue.

The energy requirement for growth was estimated following the methods of Winship *et al.* (2002). Growth requires the synthesis of protein and lipid. The energy required for growth in a given time (*E*_G_, in kilowatts) is therefore the total mass gain in lipid and protein, multiplied by their respective energy density values:

(6) $$E_{G}=dM\times [(P_{\mathrm{lip}}\times ED_{\mathrm{lip}})+(P_{\mathrm{pro}}\times ED_{\mathrm{pro}})$$

where d*M* is the change in mass due to growth in a given time period (in kilograms per second), *P*_lip_ and *P*_pro_ are the proportions of lipid and protein growth, respectively, and ED_lip_ and ED_pro_ are the energy densities of lipid and protein (in ­kilojoules per kilogram), respectively (Winship *et al.*, 2002; Fortune *et al.*, 2013).

Lipid is found in both the blubber and lean tissue of whales, in differing amounts. As we could find no lipid content values separated for blubber and muscle recorded for humpback whales, we assume that values for fin whales will be representative across large whale species (Lockyer *et al.*, 1985). The lipid content of fin whale blubber is estimated at 87%, while in muscle it is only 7% (Lockyer *et al.*, 1985). Based on the earlier mass growth values (1644 kg blubber, 7206 kg lean tissue), this calculates to 22% of growth stored as lipid (16% in blubber, 0.06% in lean tissue). As muscle was used to generalize across all lean tissue, this will be an underestimate.

Lean tissue growth is assumed as anything that is not lipid. However, lean tissue also contains water. Thus, the amount of protein in the lean tissue will be $1-P_{w}$, where *P*_w_ denotes the proportion of water in lean tissue. Therefore, Equation 5 can be rewritten to match the equation of Winship *et al.* (2002):

(7) $$E_{G}=dM\times [(P_{\mathrm{lip}}\times \;ED_{\mathrm{lip}})+(1-P_{\mathrm{lip}})(1-P_{w})\times ED_{\mathrm{pro}}]$$

Finally, the total energy demand also needs to account for the assimilation efficiency of milk, because a portion of ingested energy will be lost through faeces and urine (Winship *et al.*, 2002; Fortune *et al.*, 2013). Assimilation efficiency for preweaned blue and fin whales has been estimated as 86 and 93%, respectively (Lockyer, 1981). We therefore estimated assimilation efficiency to be 90%, meaning that required energy is 110% of growth.

The daily energy requirement of a calf (*E*_CALF_, in kilojoules per day) is summarized as:

(8) $$\begin{array}{l} E_{\mathrm{CALF}}=2E_{\mathrm{BMR}}+E_{\mathrm{COT}}+1.1E_{G}=585.8M^{0.75}\\ \;\;\;+\left(\frac{43,200\lambda }{\epsilon_{A}\epsilon_{P}}\;\right)\rho SC_{d}V^{3}+1.1dM\\ \;\;\;\;\times [(P_{\mathrm{lip}}\times \;ED_{\mathrm{lip}})+(1-P_{\mathrm{lip}})(1-P_{w})\times ED_{\mathrm{pro}}]\; \end{array}$$

## Simulation approach

The energetics model was run under four different scenarios limited to the southbound migration (breeding to feeding grounds) of mother–calf pairs, because this is when females are lactating and when whales are known to rest (Jenner *et al.*, 2001). The first two scenarios establish optimal migration energy allocation with varying average migration velocities (1) and varying rest-to travel-time ratios (2). The effects on calf growth of increased migration velocity (3) and the total migratory distance (4) are then determined (Table 2). Simulations were programmed in Python programming ­language (www.python.org).

**Table 2:** Parameters varied in each scenario to investigate how the energy use of humpback whales changes in various migration conditions

Scenario	Parameter(s) varied	Parameter range
1	Average migration velocity	0.2–4 m s^−1^
2	Travel days (to rest days)	10–90 travel days (80–0 rest days)
	Travel velocity	8500 km ÷ number of travel days
3	Average migration velocity	0.2–4 m s^−1^
4	Distance travelled	8500–10 500 km

### Scenario 1: ideal migration velocity for mothers

The ideal migration velocity was investigated using the adult female model ($E_{\mathrm{FEMALE}}=E_{\mathrm{BMR}}+E_{\mathrm{COT}}+E_{\mathrm{LAC}}$). Daily energy expenditure was recorded as the mass of blubber lost, calculated by dividing energy use by the energy density of lipid. Energy expended through *E*_BMR_, *E*_COT_ and *E*_LAC_ were recalculated each day, taking into account the mass lost by the female and mass gain of the calf from the previous day.

Average migration velocity was varied by 0.2 m s^−1^ between 0.2 and 4 m s^−1^ over a migration distance of 8500 km (Rasmussen *et al.*, 2007), to accommodate the range of swimming speeds of humpback whales, resulting in a total of 20 simulations. For each velocity, the total energy expended over the southbound migration for the female was recorded, and the proportion of total blubber used was calculated assuming a starting blubber amount according to the mass–sculp relationship of Ryg *et al.* (1993). A whale travelled at the defined speed until the 8500 km journey was completed, so that the faster the velocity, the shorter the travel time. However, given that food in the Southern Ocean is available only during the summer months, completing the migration journey early does not guarantee food availability at the time of arrival. In the model, whales must ‘wait’ to end fasting until the full 90 day migration period ends. Waiting whales were defined to be actively searching for food but not benefiting from prey acquisition. Wait velocity was assigned a value of 1.5 m s^−1^, corresponding to the slowest average foraging dive speeds recorded for humpback whales (Dolphin, 1987).

### Scenario 2: ideal resting time for mothers

Humpback whale mother–calf pairs select calm habitat in which to rest and breed (Whitehead and Moore, 1982; Craig and Herman, 2000; Ersts and Rosenbaum, 2003; Rasmussen *et al.*, 2007; Oviedo and Solis, 2008; Felix and Botero-Acosta, 2011; Braithwaite *et al.*, 2012; Cartwright *et al.*, 2012; Smith *et al.*, 2012). These habitats provide an environment where whales can rest in order to conserve energy, maximizing the allocation of energy into growth. Furthermore, the amount of resting time will also influence the rate of milk delivery. Reduced opportunities to nurse through disturbance, for example, will result in increased milk delivery rates; to maintain ideal calf growth rates, a mother will need to increase milk delivery rates to compensate for the reduced nursing time (King and Heinen, 2004; Stensland and Berggren, 2007). Thus, adopting a rest–travel strategy would benefit nursing whales by offering opportunities to nurse calves.

To test for an energetic advantage to resting, the energy use of a female whale was examined across varying rest–travel strategies over a 90 day migration; as the number of resting days increased, the number of travel days decreased accordingly. Resting was defined as slow-velocity travel (0.5 m s^−1^), in which no distance of migration was achieved (meandering in one area). Travel velocity was calculated as the speed necessary to complete the 8500 km journey across the number of travel days remaining, i.e. fewer travel days resulted in faster swimming speeds. Lactation during resting times is required to offset any mass lost by the calf during travelling and ensure the optimal growth mass of 2520 kg over the 90 days (28.5% of the 8850 kg growth mass over 10.5 months), maintaining the average rate of 28 kg day^−1^. The total mass of milk delivered during the resting period was therefore based on calf demands. The rate of milk delivery required to supply the total energy needed by the calf in the allotted resting days was also calculated and compared with large baleen whale feeding rates to test for physiological limitations in reduced resting scenarios. A total of 80 simulations were run, varying the number of travelling days between 10 and 90 days by 1 day.

We consider two potential impacts of disturbance on migration strategies in two separate ways, by measuring the proportion of ideal growth realized in a given scenario. Ideal growth is defined as 28 kg day^−1^, reaching 2520 kg over the 90 day migration. For both these scenarios, lactation (thus, the energy intake of the calf) was set to the value that supplied the energy to grow at this ideal rate when migrating at optimal velocities.

### Scenario 3: increased migration velocity for calves

If disturbance within resting areas restricts the ability or willingness of mother–calf pairs to rest, mean migration velocities would increase. We therefore increased migration velocities from 0.05 to 4 m s^−1^, over 80 simulations.

### Scenario 4: increased migration distance for calves

If disturbance from human activities results in displacement of the mother–calf pair, migration distances would increase. We therefore increased migration distance from 8500 to 10 500 km, over 201 simulations. The additional time to accommodate this longer migration distance at optimal swimming velocities was also determined.

## Results

### Ideal migration velocity for mothers

**Figure 1:**
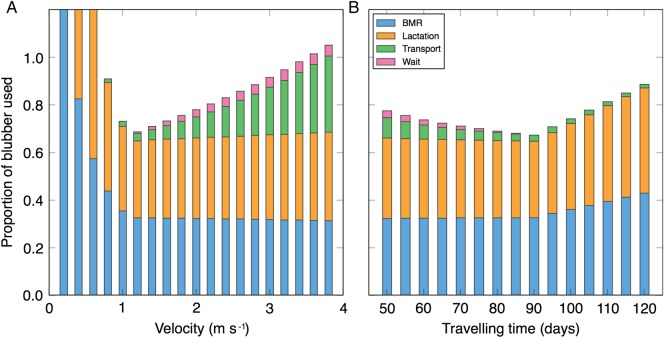
Modelled energy allocation of a lactating female whale during migration. (**A**) The proportion of blubber stores required for a lactating female to complete an 8500 km southbound migration across a range of average velocities, where food is set to appear after 90 days. If the whale completes the 8500 km in fewer than 90 days, it is assigned to ‘wait’ at 1.5 m s^−1^ for the remaining time. (**B**) The proportion of blubber stores used across the varying travelling times in the same conditions as A. Travel time is defined as the number of days to complete the 8500 km distance. The total amount of energy used has been divided into that allocated for basal metabolic rate (BMR), lactation, transport and waiting.

The energy expended for a lactating female initially decreased with increasing velocity, reaching a minimum at 1.1 m s^−1^. After this point, total energy expended increased with velocity (Fig. 1A). At slow velocities, the majority of energy expended was for BMR and lactation, with minimal transport costs and non-existent waiting costs. At high velocities, BMR and lactation remained reasonably constant, with minimal transport costs and no wait costs (because few or no waiting days occurred). This pattern of convergence to minimal energy use can also be seen across varying travel times (Fig. 1B). When the time to travel the 8500 km migration distance was 90 days (at a velocity of 1.1 m s^−1^), total energy used by a lactating female whale was minimized. A greater time spent travelling (i.e. slower velocities) resulted in greater BMR and lactation costs, while less time spent travelling (faster velocities) led to increased transport and waiting costs.

### Ideal resting time for mothers

**Figure 2:**
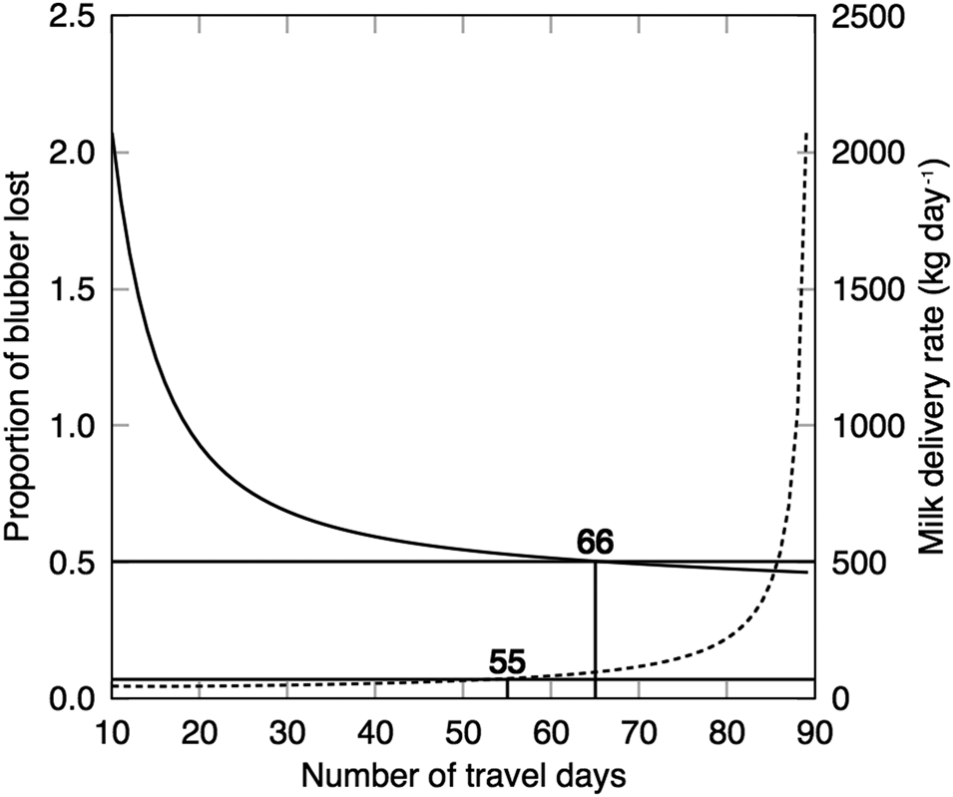
Changes in female blubber stores and lactation rate in different travel–resting regimens. Calf feeding was limited to resting days, and ideal calf growth rate of 2520 kg over 90 days was assumed, so that any increase in calf maintenance costs through travelling are met by the mother, and all energy required by the calf for growth is transferred during resting. The continuous line indicates the proportion of blubber used by the female for migration and lactation, while the dashed line is the transfer rate of milk required per day to meet the energy needs of the calf.

When the number of rest-to-travel days was varied, the energy use of whales decreased with increasing numbers of travel days (Fig. 2). When only a few days were available for travel and the whales had to swim at fast velocities to complete the migration distance in a short time, the total energy expenditure of the mother was greater than available blubber stores, indicating that the whale would perish from exhaustion. To limit energy expended to 50% of blubber reserves, a female would need to travel for 66 days, resting for 24 days. Milk delivery rate increased exponentially with increasing number of travel days (Fig. 2) due to the reduced amount of resting time; while the energy demands of the calf decreased at the slower velocities (increased travel time), this was not enough to match the reduced time available for nursing, and so milk delivery rates increase to compensate. The 24–66 day rest-to-travel ratio requires milk delivery rates of 98 kg day^−1^ in order to transfer a sufficient amount of energy to the calf over fewer nursing opportunities during fewer resting days. These delivery rates exceed the 70 kg day^−1^ milk delivery rate of fin whales (Lockyer, 1981); the model requires a female to rest for at least 35 days (and travel for 55 days) for milk delivery rates to fall below known physiological limits.

### Calf disturbance

**Figure 3:**
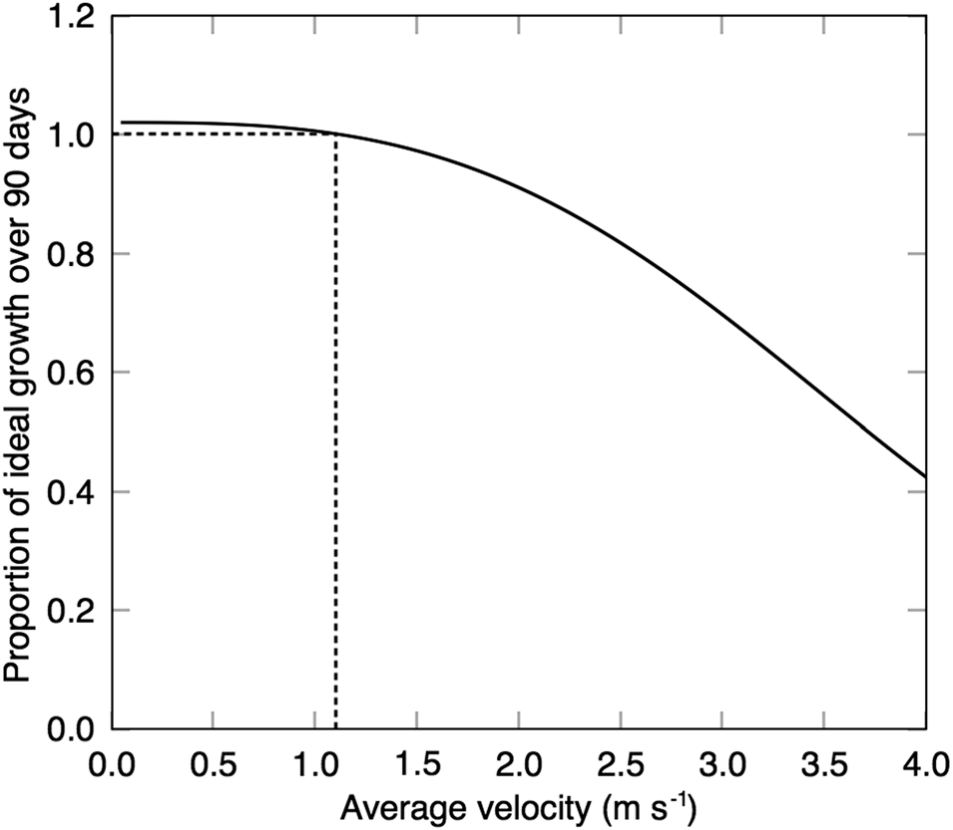
Changes in the growth of calves at increasing migration travel velocity over a 90 day migration. Ideal growth is defined as 28 kg day^−1^, and milk intake is fixed at the amount required to meet the ideal growth rate at 1.1 m s^−1^ (optimal average migration speed; see Fig. 1A).

**Figure 4:**
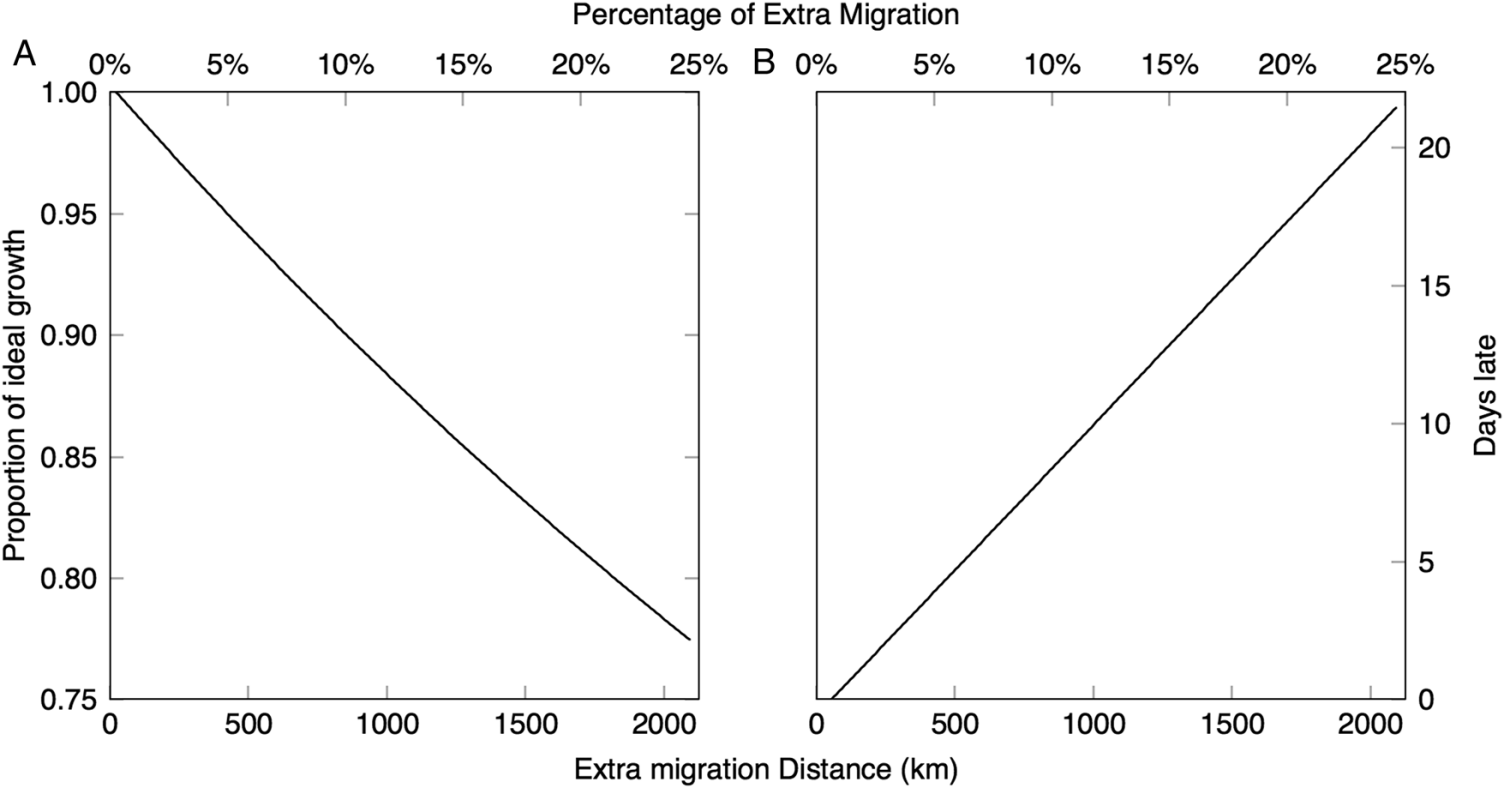
The change in calf growth rates (**A**) and number of days late to the foraging ground (**B**), as a function of increased migration distance. Ideal growth is defined as 28 kg day^−1^, and milk intake is fixed at the amount required to meet the ideal growth rate at 1.1 m s^−1^ (optimal average migration speed; see Fig. 1A). Extra distance is measured both in kilometres (marked on the bottom axis) and as a percentage of total migration (marked on the top axis).

**Figure 5:**
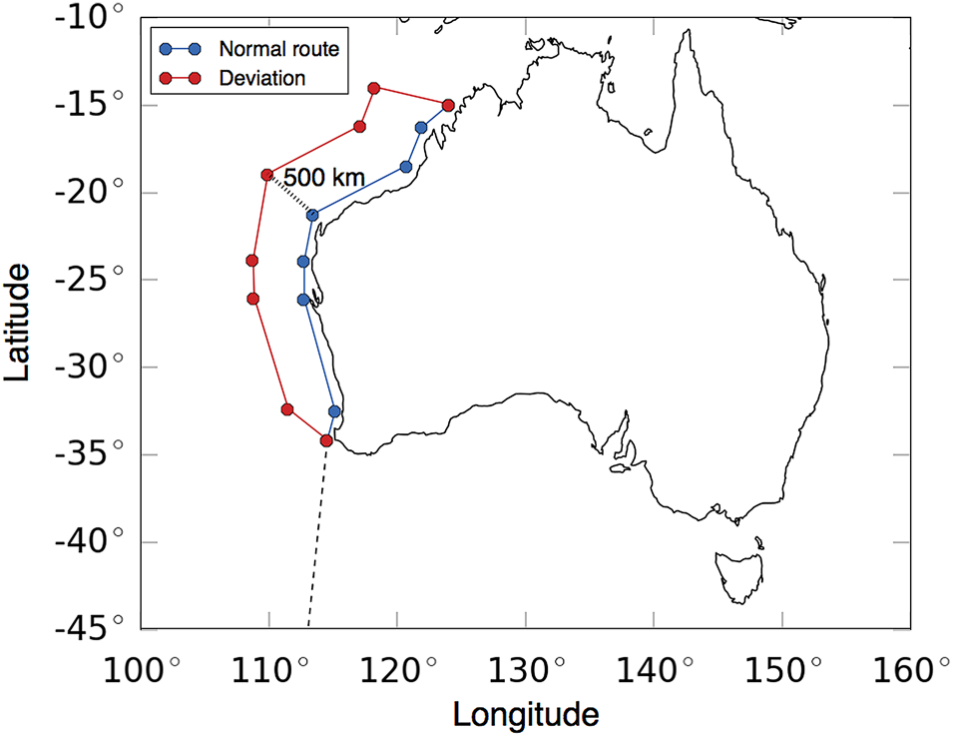
A representation of a deviated route (red) that would add ∼10% distance to a typical southbound migration journey (blue). A deviation of 500 km offshore would be required to achieve this increase in distance.

With increased migration velocity, calf growth rates decreased (Fig. 3). A doubling of average speed, from 1.1 to 2.2 m s^−1^, resulted in an 85% reduction in calf growth. With increased migration distance, ideal calf growth decreased, and the number of days whales arrived late to the foraging grounds increased (Fig. 4). For example, a migration journey with an extra 850 km resulted in a 10% reduction in calf growth, with whales arriving at the foraging grounds ∼5 days behind schedule. For this 10% reduced growth rate to occur, due to coastal disturbance, the southbound migration route would need to be diverted an extra 500 km offshore of the West Australian coast (Fig. 5).

## Discussion

Energy is an important resource for migrating whales. These capital breeders rely entirely on stored energy (blubber) to fuel long migrations in order to breed, without the option of replenishment. Budgeting energy use will therefore form a crucial component to ensure migration is completed successfully and reproductive investment maximized. In this study, we developed a theoretical bioenergetics model for migrating humpback whales to investigate the optimal migration ­strategy that minimizes individual energy use. We focus on the first 3 months of the southbound leg of the journey, between departure from breeding areas on the northwest Australian coast and arrival at the foraging grounds in the Southern Ocean. The two main factors contributing to energy conservation were the average migration velocity and the amount of time spent resting vs. travelling. Velocity, in particular, had a large influence on total transport costs. Disturbance to migration energetics changed the allocation of energy in whales; an increase in both average velocity (thereby increasing drag) and migration distance resulted in increased total energy use, impacting the growth rates of calves.

### Optimal migration

The optimal velocity to minimize energy use was a trade-off between the accumulation of daily maintenance costs at slow velocities and the expensive transport cost at high velocities. Slow migrations minimize the energy expended through transport, but accrue daily maintenance costs to support the extended journey time. Indeed, travelling too slowly will exhaust energy stores before migration is completed, resulting in mortality. The accumulation of daily maintenance costs can be reduced by travelling faster, but faster velocities incur higher transport costs due to the increase in drag.

Furthermore, food availability for humpback whales is highly seasonal, with krill biomass increasing considerably during the spring and summer seasons as a result of reduced ice cover and increased primary production in the polar region (Siegel, 2005). Consequently, reaching the foraging grounds early yields no energetic advantage for a whale, and the whale would be required to ‘wait’ for food to become available at sufficient densities to offset the high costs of ­feeding (Kenney *et al.*, 1986; Goldbogen *et al.*, 2008, 2011). Thus, daily maintenance costs are only reduced to a point, defined by the length of time until food appears.

In migrating birds, optimizing the speed of flight is also a compromise between minimizing the total duration of the migration journey and minimizing total energy expenditure (Alerstam and Lindström, 1990; Alerstam, 2011). When it is beneficial to arrive early at a destination, a faster speed to reduce migration time is advantageous, whereas if no early arrival benefit accrues, flying more slowly to reduce the total cost of flight transport is appropriate (Hedenstrom and Alerstam, 1995). It appears, from observed flight speed, that birds maximize the distance flown per unit of work done, thereby minimizing total energy costs (Hedenstrom and Alerstam, 1995). However, the deviation of measured speeds from optimal predictions varies with other factors, such as body mass (Pennycuick, 1997; Pennycuick *et al.*, 2013). Regardless, the same trade-off exists with respect to optimizing energy use by balancing the timing of migration and the cost of transport for long-distance migrators.

Travelling at an average migration speed that balances daily maintenance with transport costs not only minimizes energy use but also reduces the likelihood of mortality due to starvation *en route*. For a female travelling 8500 km in 90 days, the optimal migration velocity, at which energy was minimized, was 1.1 m s^−1^. This speed is comparable to, but a slight underestimate of observed migration velocities. Chittleborough (1953) noted that female humpback whales with calves travelled slowly, at velocities around 1.4–1.8 m s^−1^ (2.8–3.5 knots). However, these observations were made while whales were travelling between resting areas, and our model estimates the mean speed for the entire migration. Assuming a slower velocity of swimming in resting areas in comparison to travelling (Jenner and Jenner, 2011), the average velocities for the entire migration would be slower than those observed during periods of travel, accounting for the discrepancy between our model and observed values. Thus, when the distance and time of migration inputs to our model reflect conditions experienced by west Australian humpback whales, the speed at which energy use is minimized is within the realm of those observed.

When organisms divide limited resources among competing energy demands, the expected evolutionary outcome is an allocation that maximizes reproductive outcomes (Perrin and Sibly, 1993). Alerstam *et al.* (2003) reviewed several factors that may contribute to the evolution of long-distance migration strategies. For example, refuelling at stopover sites by migrating birds reduces the energy used for transport through minimizing heavy fuel load costs (Alerstam *et al.*, 2003), and migrating birds that arrive at breeding grounds with more fat stores experience greater reproductive success (Smith and Moore, 2003). For a migrating humpback whale, minimizing the energy expended for transport and maintenance enables more energy to be invested into reproductive output. Thus, the observed migration speeds of humpback whales may reflect the evolutionary selection for a migration strategy that maximizes the energy available for reproductive investment.

A second energy-saving strategy exists through optimizing the amount of time a whale spends resting vs. travelling. Given a fixed migratory duration, longer resting periods mean less time available for travel, and whales are required to swim at faster speeds to complete the migration journey, resulting in higher transport costs. Consequently, less time spent resting reduces the energy expended through transport. However, in this model we assumed that the majority of nursing occurs in resting areas, where conditions are calm and nursing efficiency can be maximized. A minimal resting period is therefore needed to transfer enough energy from mother to calf, given the limitation of milk delivery rates (Lockyer and Brown, 1981). The optimal resting period to minimize total energy use was predicted to be about 30 days (24–35 days), leaving ∼60 days (55–66 days) for travelling. Whilst humpback whales are known to aggregate in specific coastal areas for a period of time during the southbound migration (Chittleborough, 1953; Jenner *et al.*, 2001), the total amount of resting time along the entire journey remains unknown. To a calf, resting is analogous to bird stopover areas, using the area for food acquisition to fuel migration. It is currently unclear whether the rest–travel strategy is used for energy conservation in the west Australian humpback whale population. State space modelling using tracking data (e.g. Bailey *et al.*, 2009), or similar methods, may reveal behavioural budgets and how individuals divide their time during migration. The ideal time spent in stopover sites for birds is related to factors such as fuel deposition rates and distances between these sites (Alerstam, 2011). Given the increase of required milk with decreased resting time, it may be that similar optimization criteria exist in resting areas, and the ideal time spent resting will depend upon the rate of energy acquisition by the calf. Thus, the conditions of resting areas to enable efficient energy transfer from mother to calf may influence the ideal migration strategy.

### Disturbance

Both timing and energy balance are important components to successful migration and breeding, and disturbance to either of these can have repercussions for migrants. Delays to anadromous fish migrations due to river dams, for example, affect both reproductive outcomes, by changing the times and areas of spawning, and mortality rates (Castro-Santos and Letcher, 2010). Likewise, human disturbance in bird stopover sites can reduce energy gain, resulting in lower reproductive success of individuals with poorer body condition at the breeding grounds (Drent *et al.*, 2003). Human activity has the potential to disturb humpback whales during their migration, particularly when occupying shared space in coastal regions (Maybaum, 1993; Au and Green, 2000; McCauley *et al.*, 2000; Miller *et al.*, 2000; Fristrup *et al.*, 2003; Gordon *et al.*, 2003).

Here, we found that both increased velocity and lengthened migration distance reduced calf growth. Velocity is a cubed term when calculating transport costs; any rise in velocity resulting from increases in behavioural activity will have a non-linear impact on energy use and growth rates. Displacement of humpback whales from their migration path also incurred costs to energy use and calf growth. Furthermore, it delayed the arrival of migrants at the foraging grounds, potentially reducing their subsequent energy gain over the feeding season. Therefore, regardless of whether a whale is disturbed or displaced, changes to optimal migration patterns will result in higher energy demands, use of a larger proportion of blubber reserves and reallocation of energy away from growth in calves.

Early growth is an important life stage for an animal, and reduced nutrition at this time can affect long-term attributes, such as a lower adult body size and shortened lifespan (Metcalfe and Monaghan, 2001). Disturbance to calf growth during migration may therefore have long-term cross-generational implications on population status. A female could offset the reduced growth rate of a calf by supplying a greater amount of milk, meaning that disturbance would result in a 2-fold effect on a lactating female because she would need to supply the energy required to meet the increased demands of both herself and her calf, further depleting her energy reserves.

Disturbance to migrating whales can affect energy use and calf growth. However, it is still unknown whether current or predicted levels of disturbance present a significant threat to this population of humpback whales. Approximately one-third of the migration journey (∼2850 km) occurs along the west Australian coastline, with the migration path generally found to be in depths of <200 m (i.e. within the continental shelf boundary) and often around the 30 m depth contour (Jenner *et al.*, 2001). Recreational activities along the west Australian coastline, such as marine tourism, are mainly concentrated close to the shore (Hardiman and Burgin, 2010). Likewise, the main fisheries of Western Australia, western rock lobster (*Panulirus cygnus*), abalone (*Haliotis* spp*.*) and prawns (*Penaeus* spp*.*), occur in zones relatively close to the coastline and in embayments (Kangas *et al.*, 2006a, b; de Lestang *et al.*, 2012; Hart *et al.*, 2013). Human disturbance to humpback whales from recreation and fisheries would thus potentially displace migration paths only a relatively short distance offshore. Alternatively, offshore mining activities extend to 300–400 km from the shore in the Kimberley and Pilbara regions of Western Australia (Department of Mines and Petroleum, 2014). Given that our model indicates a need for a 500 km offshore displacement for a 10% reduction in calf growth, it is not clear how disruptive current human activities are to whale energetics.

Altering behavioural activity may be a more relevant consideration for whale energetics. A whale that spends 30 days resting at 0.5 m s^−1^ would be travelling at 1.6 m s^−1^ for the remaining 60 days to complete the 8500 km distance. If, for example, swimming speed in resting areas was increased to velocities comparable to travelling (1.6 m s^−1^), then calf growth would be reduced by ∼5%. This may be a more realistic scenario, given the evidence of human activity in changing the behavioural state and increasing the activity level of humpback whales through, for example, initiating avoidance behaviour and faster swimming speeds (Au and Green, 2000; McCauley *et al.*, 2000). Another possible repercussion of disturbance to resting behaviour is the reduced opportunity for nursing (King and Heinen, 2004; Stensland and Berggren, 2007). The maximal amount of milk a calf can receive is limited by the rate of delivery from the mother and the time available for feeding. Reduced resting time, and hence nursing time, means that the rate of milk delivery must increase to maintain ideal growth rates. However, milk delivery rate has physiological limits and cannot be adapted in response to the increased demands of a calf in the same way as behavioural changes can, such as more time spent nursing. If resting time is reduced to the point where the maximal rate of milk delivery is exceeded, calf growth will be compromised. Assuming that maximal milk delivery rate is 70 kg day^−1^, reducing resting time by 7 days would result in 20% less milk delivered to the calf.

Carry-over effects are an important consideration when assessing the impact of disturbance, because changes to an animal's condition can affect future performance (Harrison *et al.*, 2011); for example, foraging success in capital breeders determines body condition, which then influences reproductive success (Derocher and Stirling, 1990; Bowen *et al.*, 2001a, 2006; Wheatley *et al.*, 2006). Therefore, while the implications of disturbance to the energy balance of migrating whales may seem minor in a short-term context, these changes in energy may have long-term knock-on effects to reproductive investment. For example, breeding females are required to increase body weight by 65% in order to fuel migration and breeding, as opposed to ∼50% for other non-breeding whales (Lockyer, 1981). These high energy demands in female baleen whales can necessitate a ‘rest’ year from breeding if stores are not replenished over the foraging season (Lockyer, 1986; Williams *et al.*, 2013). Additional migratory energy demands for the female could lengthen the replenishment period. Reduced calving rates will have implications for population growth rates. Additionally, the body condition of humpback whales fluctuates with annual changes in food abundance (J. E. Braithwaite, J. J. Meeuwig and M. R. Hipsey, unpublished work), influencing the initial capital able to be invested for migration. In food-poor years, whales will therefore be migrating on lower energy stores, and suboptimal expenditure during migration, perhaps induced by disturbance, will have a greater effect on the proportion of stores used. Extension of the model developed here to include both variations in initial body condition and measures of fecundity would enable the investigation of carry-over effects between seasons and years.

Another component to energy use not considered in this model is hydrodynamic condition, which may factor into the energetic repercussion of course diversion. Current velocity and flow direction affect the drag forces on a whale, and swimming velocity will need to be adjusted accordingly to maintain a particular speed over ground (McElroy *et al.*, 2012). The hydrodynamic conditions encountered by a whale can therefore have either a positive or a negative influence to the cost of transport, depending on whether they are swimming with or against the flow of water, and the strength of flow encountered (Bose and Lien, 1990). Migrating anadromous fish, for instance, select paths up river that are significantly less energetically demanding than random path conditions, a behaviour interpreted to conserve transport costs and allow for more energy to be invested into reproduction (McElroy *et al.*, 2012). Likewise, birds exploit favourable wind conditions to economize energy use (Liechti, 2006). Indeed, extreme bird migrations across the Pacific Ocean use a wind-assisted corridor resulting from global atmospheric circulation in order to travel these large distances (∼10 000 km) without stopping (Gill *et al.*, 2009).

It may be that migrating humpback whales also use hydrodynamically favourable paths to reduce transport costs. In west Australia, the southward flowing Leeuwin current creates generally favourable flow conditions for southbound migrating humpback whales in the continental shelf region (Feng *et al.*, 2003). While the flow is weaker during spring and summer, when whales are travelling south, diversions of 90 km offshore (Pearce and Pattiaratchi, 1999) would be required to redirect whales outside these favourable conditions. Therefore, while a small diversion off course has a seemingly minimal impact on energy use in terms of extra distance to travel, it may divert whales to less hydrodynamically favourable conditions, increasing the amount of energy expenditure in an unknown way that our model does not consider.

### Model sensitivity and uncertainty

The absence of empirical information regarding humpback whale physiology led to a number of assumptions regarding migration energetics and parameter estimation. First, we measured the energy expended for all physical activity in the cost of transport equation (Equation 3). Humpback whales display a variety of behaviours, ranging from passively lying at the surface to more active tail slapping and breaching (Corkeron, 1995). These behaviours will have different energetic costs from the constant swimming motion used in our model. As unsteady movement patterns incur extra energy expenditure (Daniel, 1985; Fish, 1994), we are likely to be underestimating the energy used for increased behavioural activity. However, as active behaviours typically involve more physical movement than passive behaviours, grouping all activity as the cost of transport provides a general indicator of how changes in behaviour may affect overall energy use.

A second assumption made was the linear rate of growth in a calf. Growth is generally a curved function, beginning relatively fast and slowing towards maturity (Bertalanffy, 1938), and large whales are no exception (Lockyer, 1981; Fortune *et al.*, 2012). To our knowledge, humpback whale growth curves have been calculated only with regard to length, rather than mass, so we were unable to integrate a mass growth rate function into this model. However, age–length relationships show growth to be relatively constant for the initial months, slowing at 1 year of age (Stevick, 1999). We therefore assumed that uncertainty from the use of a linear growth model is ­minimal; however, any extension of this model to include growth beyond weaning will need to account for a curved growth function in calves.

Third, energy provided from lactation was assumed to be constant in our model, although the proportion of milk constituents in cetaceans changes over time (Oftedal, 1997). In humpback whales, for example, the proportion of fat increases over the first few months of lactation, before decreasing again towards weaning (Oftedal, 1997). As we used the 4–7 month average proportion of constituents, the energetic value of milk would be greater than that of early months, and thus, the total amount of milk required to support ideal growth will be an underestimate. Consequently, the amount of resting time, as predicted by milk transfer rates, will also be underestimated.

Fourth, the states of resting and waiting were categorized by velocity, set respectively at 0.5 and 1.5 m s^−1^, and in the third scenario (disturbance to calf growth) the travelling velocity was set at 1.1 m s^−1^. However, given that velocity is defined as a cubed term in the model when calculating the energetic cost of transport, the cost of transport is highly sensitive to small changes in these set velocity parameters. We used conservative velocity estimates, at the lower end of estimated resting and foraging speeds, so the cost of transport calculated by our model will probably be an underestimate. However, obtaining and integrating telemetry data into the model analysis would be beneficial to obtain better estimates of velocity values in different behavioural states.

Many parameters are not well defined for humpback whales in the literature, and values for other species were used instead, adding to the uncertainty of model outputs. Where possible, we obtained parameter values for large whale species to minimize this uncertainty. Tortuosity, the deviation away from a straight-line path, was one parameter that had not been defined for travelling humpback whales and therefore not included in our model. Thus, the raised transport cost associated with not travelling in a straight line was not accounted for in our model, and the resulting energy use associated with transport costs calculated by our model will be underestimated. Again, analysis of telemetry data would be advantageous to defining this parameter, so that the cost of transport can be better estimated.

### Conclusions

Whales migrate large distances on a limited energy budget, and managing this energy budget optimally is important to ensure survival over the migration route and to maximize reproductive output. The theoretical model developed in this study demonstrated that an optimal migration strategy exists, in which energy use is minimized through management of both swimming velocity and the time spent resting. At each end of the scales, greater than available reserves were required, which would lead to the exhaustion of stores before migration was completed. Observed swimming velocities of migrating whales were comparable to those predicted by the model, suggesting that minimizing energy use during ­migration may be a contributing factor to the evolution of observed migration patterns. Furthermore, human disturbance along the migration route has the potential to alter the energy budgets of these animals by increasing the total energy required and reducing the amount available to be invested in calf growth. Resting areas are particularly vulnerable to disturbance, because disruption to resting behaviour can impede both the amount of milk transferred to the calf and the proportion of this milk allocated to growth. While further developments are necessary to determine the long-term repercussions of migration disturbance to reproductive outcomes and population growth rates, this model provides initial insights into the energy trade-offs associated with migrating and breeding for baleen whales.
